# Do cows with stereotypic tongue-rolling behaviour cope better with their environment?

**DOI:** 10.3389/fvets.2024.1404539

**Published:** 2024-05-22

**Authors:** Chenyang Li, Xiaoyang Chen, Tingting Fang, Xianhong Gu

**Affiliations:** ^1^State Key Laboratory of Animal Nutrition and Feeding, Institute of Animal Science, Chinese Academy of Agricultural Sciences, Beijing, China; ^2^College of Animal Science and Technology, China Agricultural University, Beijing, China

**Keywords:** animal welfare, stereotypic behaviours, tongue-rolling, stress, lactation performance

## Abstract

**Introduction:**

Stereotypic behaviours, especially oral stereotypic behaviours, are frequently expressed in farm animals. Tongue-rolling is the most common oral stereotypic behaviour in dairy cows (*Bos taurus*). If animals frequently display stereotypic behaviours, this is an indication of poor welfare. It has been suggested that animals express stereotypic behaviours as a way of coping with stress. As a result, animals with stereotypic behaviours may have lower levels of stress hormones than animals without stereotypic behaviours.

**Methods:**

In this study, 916 Holstein cows in the first lactation were subjected to scan sampling behavioural observations 200 times for 10 days. All cows were assigned to either a stereotypic behaviours group (SB) or a control group (CON). The SB group was further subdivided into a tongue-rolling group (TR) and an other-stereotypic behaviours group (OS). The TR group was also split into an only tongue-rolling group (OTR) and a mixed tongue-rolling and other stereotypic behaviours group (TROS). Some cows in the TR group belonged to an extreme tongue-rolling group (ETR). Hair and saliva samples were collected from 601 cows to test cortisol concentrations and dairy herd improvement (DHI) data were collected from a total of 762 cows.

**Results:**

There were no differences in hair or saliva cortisol concentrations between the groups (*p*>0.05), and the frequencies of tongue-rolling were not associated with cortisol concentrations (*p*>0.05). For DHI in cows, the milk protein percentage (*p* = 0.028), milk true protein percentage (*p* = 0.021) and milk crude protein percentage (*p* = 0.023) of cows in the ETR group were significantly lower than those in the CON group. For cows in ETR group, as the frequencies of tongue-rolling increased, the milk protein percentage (*p* = 0.034, r = 0.365), milk true protein percentage (*p* = 0.022, r = 0.393) and milk crude protein percentage (*p* = 0.035, r = 0.363) increased.

**Discussion:**

We investigated the relationship between stereotypic behaviours and stress by using a non-invasive sampling method to minimise harm to the cows. We suggest that tongue-rolling may not be a way for cows to cope with stress, at least in terms of cortisol concentrations.

## 1 Introduction

**Figure 1:**
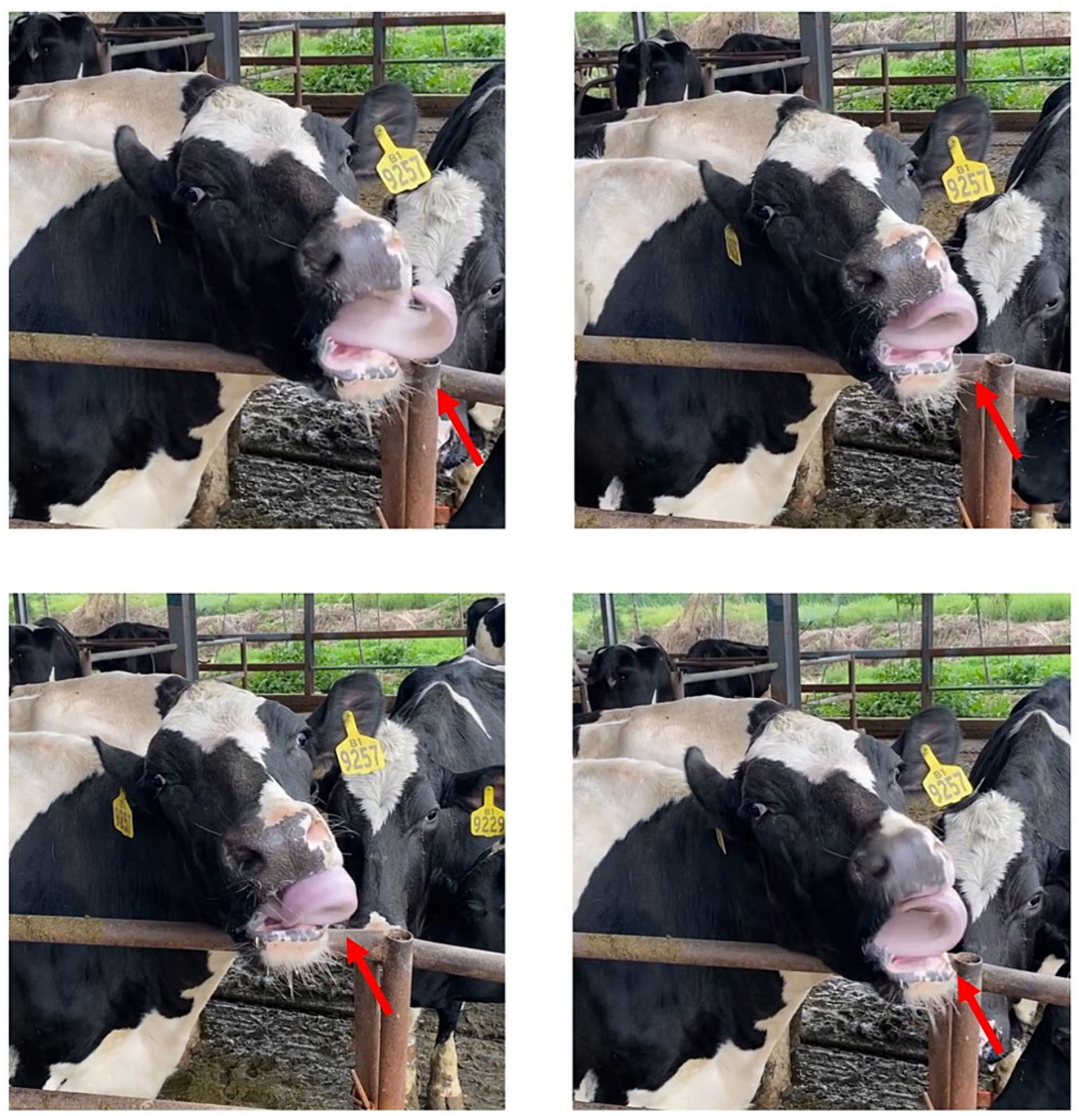
The tongue-rolling behaviour of cows. The arrow indicates the tongue of a Holstein cow. As shown in the figure, the tongue curls outside the oral cavity and makes circular movement.

Stereotypic behaviours are continuous, repetitive and seemingly non-functional behaviours that occur in farm animals, companion animals, and zoo animals (1–4). It is generally accepted that stereotypic behaviours usually occur in barren environments (5). Tongue-rolling is the repeated circular movement of the tongue inside and outside the mouth (Figure 1) and it is the most classic and common of stereotypic behaviours in cows (*Bos taurus*) (6). In a recent observational experiment on dairy cows, tongue-rolling was found to occur in 29% of cows (2,365/8158) and that the percentage of cows with tongue-rolling increased and then decreased with lactation stage (7). Prolonged stress (8), unsatisfactory foraging behaviour (9) and gastrointestinal distress (10) have the potential to cause tongue-rolling behaviour in dairy cows. For these reasons, measures to reduce tongue-rolling behaviour have also emerged, such as increasing the forage content of diets (11–13).

Animals may be chronically stressed if they live in poor environments (14). Some researchers have suggested that stereotypic behaviours may be a way for animals to cope with stress, known as the “coping hypothesis” (15). Therefore, certain individuals with stereotypic behaviours may have higher production or reproduction performance. For example, striped mice (*Rhabdomys pumilio*) expressing stereotypic behaviours have higher reproduction performance than striped mice without stereotypic behaviours (16); cows with stereotypic behaviours also produce more milk than normal cows (10). When animals are stressed, the hypothalamic–pituitary–adrenal (HPA) axis and locus coeruleus-norepinephrine (LC-NE) axis in the organism are activated, as evidenced by a rapid increase in the concentrations of glucocorticoids (e.g., cortisol and corticosterone) and catecholamines (e.g., dopamine) in the body (17, 18). In mammals, the concentration of cortisol is often used as an indicator of stress in the body (19). However, studies on stereotypical behaviours in different animals in recent decades have yielded conflicting results: (1) Animals displaying stereotypic behaviours have higher plasma cortisol concentrations than normal animals (4, 20); (2) The expression of stereotypic behaviours does not correlate with salivary cortisol concentrations (2, 21); and (3) Animals expressing stereotypic behaviours have reduced plasma or salivary cortisol concentrations (22, 23). There may be several reasons leading to varying results in research studies. The levels of cortisol in animals exhibit circadian rhythms (24), and extended sampling durations within the same experiment can result in significant variations in cortisol levels among individual animals. Additionally, the differing tolerance levels of animals to sampling methods may also contribute to the inter-individual differences in cortisol levels.

Blood, urine, feces and saliva are the main biological samples used to measure cortisol concentrations. Among them, measuring the cortisol level in blood is the most commonly used method, but the blood collection process can cause adverse stress responses in animals (25). Many studies have used saliva to assess cortisol responses and have proven this method to be feasible (26–28). The saliva collection process is a non-invasive technique that has less impact on animals and does not cause severe stress responses (29). Salivary cortisol concentration has been widely used in welfare studies of cows and pigs, and is an ideal indicator for assessing the stress conditions of cows and pigs (30, 31). In recent years, researchers have found that the long-term stress status of animals can be determined by assessing hair cortisol concentrations (32–34) and that hair samples have the advantage of being easy to collect and harmless to the animals. The relationship between the HPA axis and hair cortisol concentrations has been demonstrated in dairy cows and calves (35, 36). Hair cortisol content is at higher concentrations when cows are under chronic stress (37), such as pregnancy (38), environmental changes (39), high milk production (40), etc. Measuring cortisol concentrations in hair is a non-invasive technique to assess chronic stress in dairy cows and has been applied to assess the level of cow welfare (37).

Based on the above, the aim of our study was to investigate whether there is a relationship between stereotypic behaviours and stress status in dairy cows. We explored the relationship between stress and tongue-rolling behaviour in dairy cows by observing their behaviour and measuring the cortisol concentration in their hair and saliva. The following hypotheses were proposed: 1. If the expression of stereotypic behaviours is a way for animals to cope with stress, the salivary and hair cortisol concentrations of cows with stereotypic behaviours may be lower than those of normal cows; 2. If the expression of stereotypic behaviours is a way for animals to cope with environmental stress, the frequencies of stereotypic behavioural expression may have a correlation with hair and salivary cortisol concentrations; 3. The expression of stereotypic behaviours by animals may affect animal production performance. In addition, we researched whether the prevalence of tongue-rolling varies with the lactation stage.

## 2 Materials and methods

### 2.1 Animal welfare statement

The experiment was performed at the dairy farm of the Shandong Yinxiang Weiye Group Company (Cao County, Shandong, China, 115°26′E, 34°50′N). The farm keeps Holstein cows for milk production. All experiments were approved by the Animal Ethics Committee of the Chinese Academy of Agricultural Sciences (Beijing, China, approval number IAS2023-68). The experimental dates were from April 18, 2023 to May 5, 2023.

### 2.2 Animal, diet and management

A total of 916 cows, all in first lactation, were kept in 3 large ventilated free-stall cowsheds. On average, there were 306 cows per cowshed. Every cowshed had two pens, and every pen had 200 stalls. The cowsheds were equipped with fans and sprinklers for cooling (temporarily not utilized during the period of behavioural observations). The cows were fed the total mixed ration (TMR, 08:30, 15:30, and 20:30, dietary ingredients are described in Table 1) each after being milked (08,00, 15:00, and 20:00) three times per day. Each milking round lasted 20 min. The cowsheds were equipped with an automatic manure scraper system, and recycled manure solids were used as bedding for dairy cows. The bedding was replaced one day before the start of the experiment. The cowsheds were disinfected thoroughly once a week to ensure its hygiene and cleanliness. The health status and body condition score (BCS, scoring range of 1 to 5) of the cows were assessed weekly by a veterinarian, and the daily milk yield and days in milk (DIM) of lactating cows were automatically recorded every day by a fully automatic milking system.

**Table 1:** Ingredients and nutrient composition of experimental diet (%, DM basis).

Items	Value
**Ingredients**	**Content, %**
Alfalfa	10.39
Oat hay	2.42
Dandelion	0.48
Whole corn silage	48.33
Cottonseed	2.90
Beet pulp	2.42
Ground corn	7.49
Pressed corn	9.42
Soybean meal	8.70
Rapeseed meal	1.69
DDGS^a^	0.72
Extruded soybean	1.33
Mineral and vitamin mix^b^	3.70
**Nutrient composition**	
DM, % of wet TMR	62.40
CP	17.06
EE	3.32
NDF	35.75
ADF	18.20
NEL/(MJ/kg)	6.11

a DDGS, Distillers Dried Grains with Solubles.

b Contained the following per kg of diets: VA 170,000 IU, VD 8,000 IU, VE 9,000 IU, Ca 160 g, Fe 800 mg, Cu 680 mg, Mn 3,500 mg, Zn 7,500 mg, Se 80 mg, I 400 mg, Co 38 mg.

### 2.3 Behavioural observations and sample collection

All cows raised in the 3 cowsheds, totaling 916, were observed in this experiment. During the period of behavioural observations, no cows were removed. We used scan sampling behaviour observations by 3 well-trained observers. Before the formal observations, all observers conducted a three-day preliminary observations assessment in the same cowshed and then the prevalence-adjusted bias-adjusted Kappa (PABAK) values were calculated to be greater than 0.8, indicating that the three observers’ assessments of stereotypical behaviour in cows were almost consistent. Cows were observed daily from 08:00 to 11:10 and from 14:00 to 17:10 for 10 min each time. The observations were conducted 10 times in the morning and 10 times in the afternoon every day, with a 10 min interval between two observations, which were scheduled for 10 days (April 21 to 30) to total 200 behavioural observations. The three observers were assigned to three different cowsheds, ensuring that each cowshed had one observer. Each observer switched to a different cowshed after completing 1 day of observation and repeated the cycle every 3 days. Each observer observed all cowsheds every 3 days. In each round of scan sampling behaviour observation, an observer slowly walked from one end of the cowshed to the other to observe all the cows within the same cowshed. For each observation, if a cow exhibited some stereotypic behaviour, it was recorded as one occurrence of the stereotypic behaviour. Simultaneously, the ID of cows exhibiting stereotypic behaviours and the types of stereotypic behaviour (tongue-rolling, feed-tossing, pica, excessive-grooming, excessive-rubbing, excessive-vocalising, inter-sucking, head-shaking) were recorded. The description of the stereotypic behaviours is shown in Table 2. In our observations, when the duration of grooming, rubbing or vocalising behaviour exceeded 10 s, it was, respectively, recorded as excessive-grooming, excessive-rubbing or excessive-vocalising.

**Table 2:** The description of the stereotypic behaviours.

Behaviours	Definition
Tongue-rolling	The cow’s tongue makes repeated circular movements inside and outside the mouth.
Pica	The cow licks or bites non-food objects (e.g., fences, food trough).
Excessive-grooming	The cow grooms itself in the same area for more than 10 s.
Feed-tossing	The cow picks up a mouthful of feed and throws it into the air.
Inter-sucking	The cow sucks on its own or other cows’ body parts.
Head-shaking	The cow makes its head move quickly from side to side.
Excessive-vocalising	The cow keeps vocalising for more than 10 s.
Excessive-rubbing	The cow chafes its head or parts of its body against cowshed structures or equipment for more than 10 s.

We started collecting hair and saliva samples from cows across the same period the behavioural observations were conducted. Before the experiment, we planned to collect hair and saliva samples from 600 cows (actually collected from 601 cows), so about 2/3 of the cows in each pen were sampled. Sampling was done before the morning feeding, and lasted for 1 hour each day. We randomly collected hair and saliva samples from about 40 cows per day in the same cowshed, and sequentially changed the sampling cowshed daily among the 3 experimental cowsheds. The whole sampling lasted for 15 days. Therefore, the sampling continued for 5 days after the behavioural observations ended. After each cow was sampled, we marked it with a red crayon to prevent repeated sampling. Hair samples were collected from 3 cm from the tail switch close to the skin for hair cortisol detection. The saliva collection process was as follows: a sterile gauze was wrapped around one end of a wire, and the other end of the wire was held by hand to slowly insert the gauze into the cow’s mouth. After the cow has fully chewed for 1 to 2 min, the gauze was taken out and placed in a sterile syringe, and the saliva was squeezed into a 2 mL cryotube, which was stored in liquid nitrogen for the detection of cortisol content. As non-invasive techniques, the collection of hair and saliva samples did not elicit strong reactions from any of the cows.

According to customary practice, the dairy farm veterinarian conducted monthly Dairy Herd Improvement (DHI) tests on all lactating cows, which included measuring the milk protein percentage, milk fat percentage, milk lactose percentage, milk crude protein percentage, milk true protein percentage, urea nitrogen and freezing point. Fortunately, during the period of behavioural observations, the veterinarian carried out a DHI test. The specific process for the collection of milk samples was as follows: milk samples were collected at a volume ratio of 4:3:3 corresponding to the 08:00, 15:00, and 20:00 milking in 100 mL plastic vials. Samples were preserved with 2-bromo-2-nitropropan-1,3-diol and stored at 4°C.

### 2.4 Cortisol concentration test

Salivary and hair cortisol were detected in this experiment. Salivary cortisol concentrations were detected by radioimmunoassay. The kits were provided by Beijing Laibertaire Technology Development Co. The detection instrument was BMF-96 (Hefei Zhongcheng Mechatronics Technology Development Co., Ltd., Hefei, China). The detection limit is 1 ng/mL ~ 500 ng/mL.

We measured hair cortisol levels following the method described by Burnett et al. (41). Hair cortisol concentrations were measured by enzyme-linked immunosorbent assay (ELISA), the kits were provided by Shanghai Hengyuan Biotechnology Co. The detection limit of the kits is 5 μg/L ~ 160 μg/L.

### 2.5 Statistical analysis and grouping methods

Upon the completion of the 10-day behavioural observations, we performed statistical analysis on the behavioural data using Excel (Microsoft, WA, United States). We tallied the number of cows exhibiting tongue-rolling, feed-tossing, excessive-rubbing, excessive-grooming, pica, head-shaking, inter-sucking and excessive-vocalising behaviours, and calculated the percentages of cows with different stereotypic behaviours relative to the total herd and relative to all cows with stereotypic behaviours.

When analysing the data, we divided these cows into different groups according to different behaviours. Firstly, we divided the cows into a stereotypic behaviours group (SB, including all the stereotypic behaviours) and a control group (CON) based on their expression or non-expression of stereotypic behaviours. Secondly, we divided the cows in the SB group into a tongue-rolling group (TR, some cows in the TR group exhibited both tongue-rolling and other stereotypic behaviours) and other stereotypic behaviours group (OS, cows in OS group only exhibited other stereotypic behaviours except tongue-rolling) based on whether they expressed tongue-rolling behaviour. Additionally, we combined the OS and CON groups into the non-tongue-rolling group (NTR). Thirdly, we divided the cows in the TR group into a group only with tongue-rolling behaviour (OTR) and a group expressing both tongue-rolling and other stereotypic behaviours (TROS).

Different parts of this experiment included different sample sizes. A total of 916 cows were observed in the behavioural observations; DHI data were collected from 762 cows (Failure to collect DHI data from all cows due to errors in the sampling process or farm management); hair and saliva samples were collected from 601 cows. Previous research (42) had suggested the coefficient of variation (CV) was helpful to describe noteworthy behaviours, thus CV of tongue-rolling behaviour was calculated using the formula $CV=\left(\frac{SD}{\mathrm{mean}}\right)\times 100\%$ . By calculating the CV value (110.15%), we selected cows with a number of tongue-rolling that was greater than 1.5 times the number in the first quartile as the cows with extreme tongue-rolling group (ETR). Fortunately, all cows in the ETR group only expressed tongue-rolling and no other stereotypic behaviours.

Cortisol data and lactation performance data (included DHI data) were analysed using the SPSS 26.0 software (SPSS, Chicago, IL, United States). The information of BCS and DIM of lactating cows on the day before the start of the behavioural observation were included as categorical and continuous covariates, respectively, in the statistical analysis of each cortisol concentration and lactation performance variable. To explore the relationship between the prevalence of tongue-rolling behaviour and the stage of lactation, we divided lactation into five stages based on the DIM: 20 days and below is first 20 days, 21 to 150 days is early lactation, 151 to 210 days is mid lactation, 211 to 305 days is late lactation and after 305 days for some reason not in dry milk is over 305 days. We calculated the milk protein yield, milk crude protein yield, milk true protein yield, milk fat yield and milk lactose yield based on DHI data. Based on the formula for 4%Fatcorrectedmilk=0.4×milkyield+15×milkfatyield , calculated the 4% fat corrected milk (4% FCM). We conducted normality tests on all data. Data not conforming to normality (including hair cortisol, salivary cortisol, milk fat percentage, milk lactose percentage, urea nitrogen and freezing point) were analysed by Mann–Whitney U tests (2 groups) and Kruskal-Wallis H tests (over 2 groups). The aforementioned data are presented visually in the form of figures. Other data (milk protein percentage, milk crude protein percentage, milk true protein percentage, milk yield, milk protein yield, milk lactose yield, milk fat yield, milk crude protein yield, milk true protein yield and 4% FCM), conforming to normality, were analysed by ANOVA tests and Student’s *t*-tests. To accomplish post-hoc pairwise comparison, multivariate Duncan’s tests were used. The results are presented as the mean values and SEM.

Spearman correlation analysis was applied to determine the relationships between the frequencies of tongue-rolling expressions characteristics and hair and salivary cortisol concentrations and lactation performance. The frequencies of tongue-rolling behaviour are the percentage of the number of tongue-rolling expressions out of the total number of observations, where the total number of observations is 200. *p*-values < 0.05 were considered statistically significant.

## 3 Results

### 3.1 Behavioural observations

**Figure 2:**
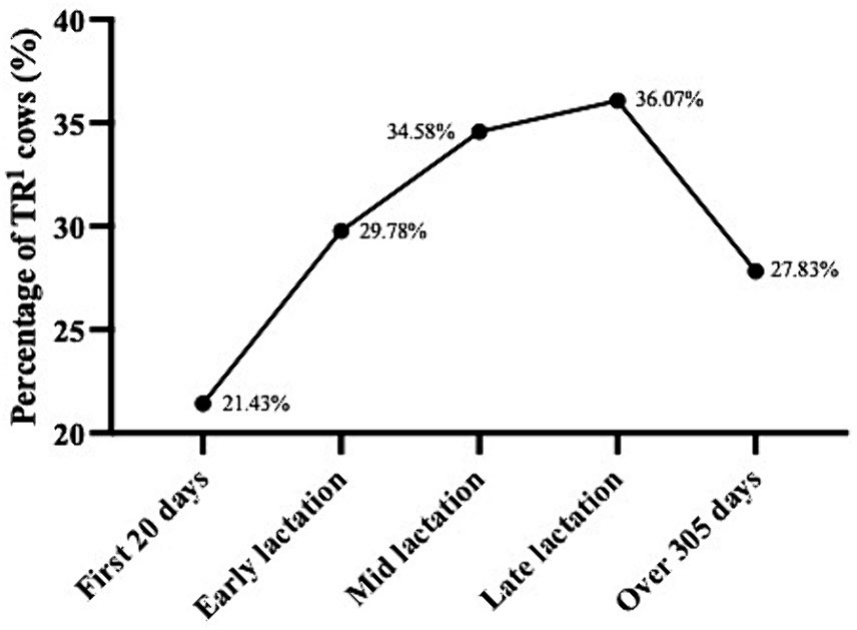
Prevalence of tongue-rolling behaviour in different lactation stage (*n* = 916). First 20 days: the first 20 days of lactation; Early lactation: days in milk from 21 to 150 days; Mid lactation: days in milk from 151 to 210 days; Late lactation: days in milk from 211 to 305 days. ^1^TR: Tongue-rolling.

In the present behavioural observations, 45.74% (419/916) of the dairy cows exhibited various stereotypic behaviours (Table 3). This included 295 cows with tongue-rolling behaviour, 71 cows with feed-tossing behaviour, 57 cows with excessive-rubbing behaviour, 43 cows with excessive-grooming behaviour, 50 cows with pica behaviour, 7 cows with head-shaking behaviour, 5 cows with inter-sucking behaviour and 4 cows with excessive-vocalising behaviour. Cows with tongue-rolling behaviour accounted for 70.41% of all stereotypic behaviours, which also indicated that tongue-rolling was the most common stereotypic behaviour in cows. The highest number of tongue-rolling expressions was 28, and 147 cows were observed with only one tongue-rolling behaviour in 200 behavioural observations. Therefore, we chose tongue-rolling behaviour for the analysis of cortisol data and lactation performance data. The prevalence of tongue-rolling behaviour increased and then decreased with DIM. The prevalence of tongue-rolling peaked in late lactation at 36.07%, while the lowest prevalence of tongue rolling was found in the first 20 days of DIM at 21.43%. The proportions of tongue-rolling cows in each different lactation stage are shown in Figure 2.

**Table 3:** Prevalence of different stereotypic behaviours (*n* = 916).

Items	Stereotypic behaviours						Total
	Tongue-rolling	Feed-tossing	Excessive-rubbing	Pica	Excessive-grooming	Others^a^	
Number of cows	295	71	57	50	43	16	419
Percentage^b^ (%)	32.21	7.80	6.22	5.46	4.70	1.75	45.74
Percentage^c^ (%)	70.41	16.95	13.60	11.93	10.26	3.82	100

The “*n*” in the table heading represents the number of cows.

a Mean other stereotypic behaviours, including inter-sucking, head-shaking, excessive-vocalising.

b This is the percentage of cows with different stereotypic behaviours relative to the total herd.

c This is the percentage of cows with different stereotypic behaviours relative to all cows with stereotypic behaviours.

### 3.2 Cortisol concentration

**Figure 3:**
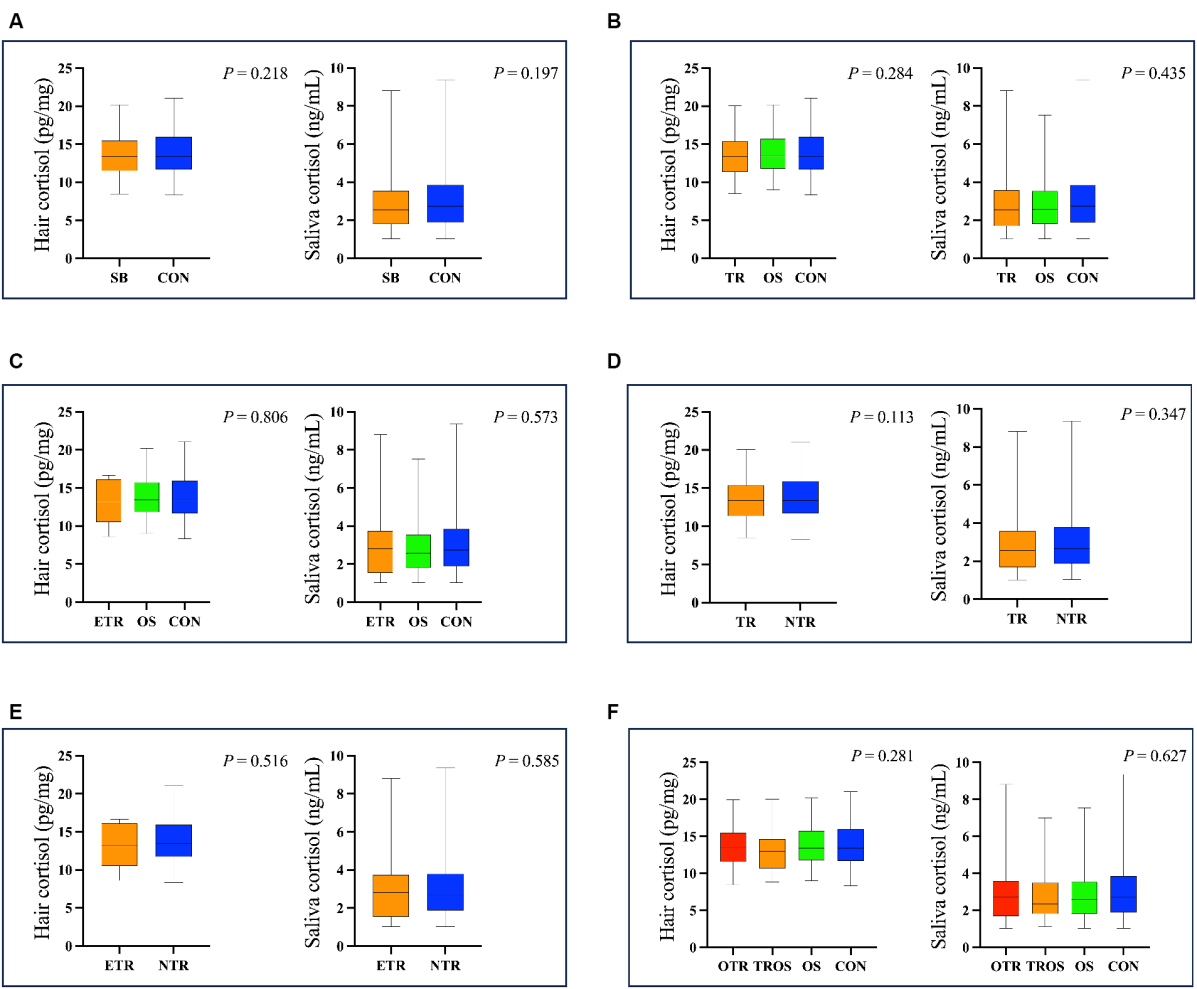
The results of hair and salivary cortisol concentration in different cows. **(A)** The cows were divided into SB group (The group of 264 cows with stereotypic behaviour) and CON group (The group of 337 cows without stereotypic behaviours). **(B)** The cows were divided into TR group (The group of 180 cows with tongue-rolling behaviour), OS group (The group of 84 cows with stereotypic behaviours except tongue-rolling) and CON group. **(C)** The cows were divided into ETR group (The group of 20 cows with extreme tongue-rolling behaviour. We selected cows with a number of tongue-rolling that was greater than 1.5 times the number in the first quartile as the cows with extreme tongue-rolling behaviour), OS group and CON group. **(D)** The cows were divided into TR group and NTR group (The group of 421cows without tongue-rolling behaviour). **(E)** The cows were divided into ETR group and NTR group. **(F)** The cows were divided into OTR group (The group of 141 cows only with tongue-rolling behaviour), TROS group (The group of 39 cows with tongue-rolling behaviour and other stereotypic behaviours), OS group and CON group.

The hair cortisol concentrations ranged from 8.38 to 21.08 pg./mg and the salivary cortisol ranged from 1.02 to 9.35 ng/mL. As shown in Figure 3, there was no difference (*p* > 0.05) in hair and salivary cortisol between SB and CON groups. When we considered tongue-rolling behaviour, there were also no differences in hair and salivary cortisol concentrations in any of the groups, even when only the ETR group was considered (*p* > 0.05, Figure 3).

**Figure 4:**
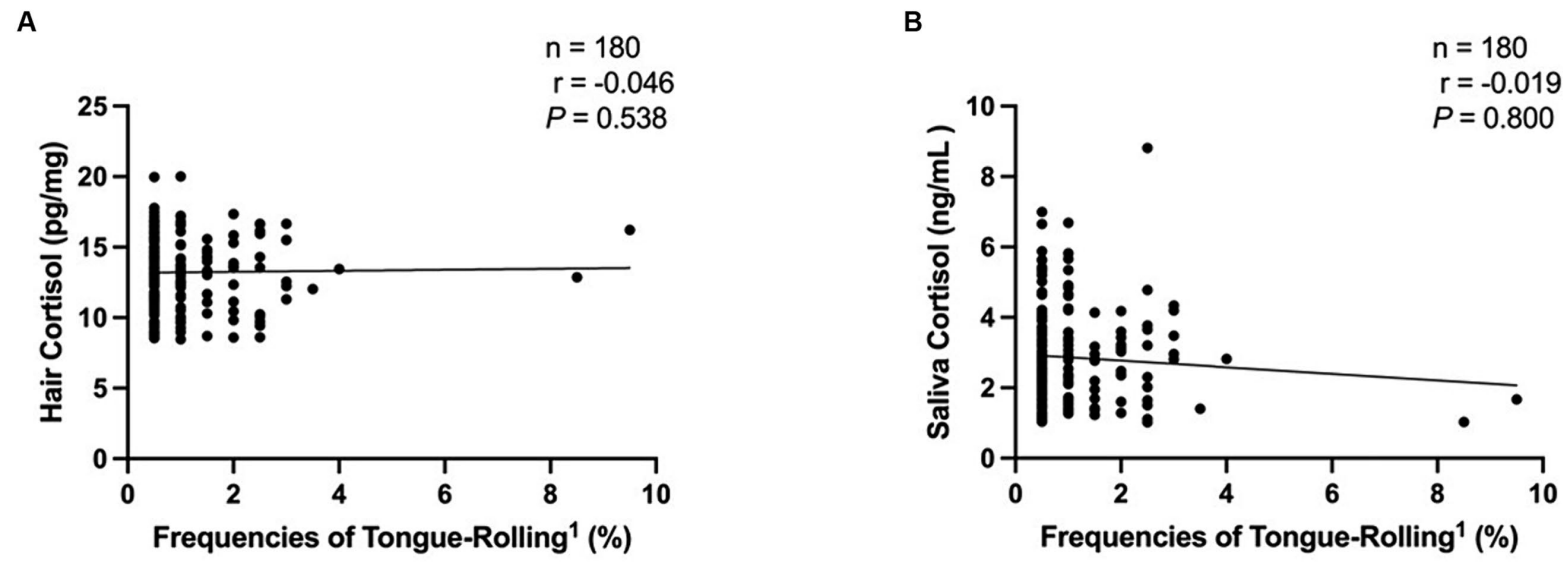
Analysis of the correlation between tongue-rolling behaviour and hair and salivary cortisol concentrations in dairy cows (*n* = 180). Among all the tongue-rolling cows, there were 180 cows collected for hair and saliva samples to measure cortisol concentration. **(A)** The analysis of the correlation between tongue-rolling behaviour and hair cortisol concentrations. **(B)** The analysis of the correlation between tongue-rolling behaviour and saliva cortisol concentrations. ^1^Frequencies of Tongue-Rolling = the percentage of the number of tongue-rolling expressions out of the total number of observations for cows exhibiting tongue-rolling with cortisol concentration measured, where the total number of observations is 200.

**Figure 5:**
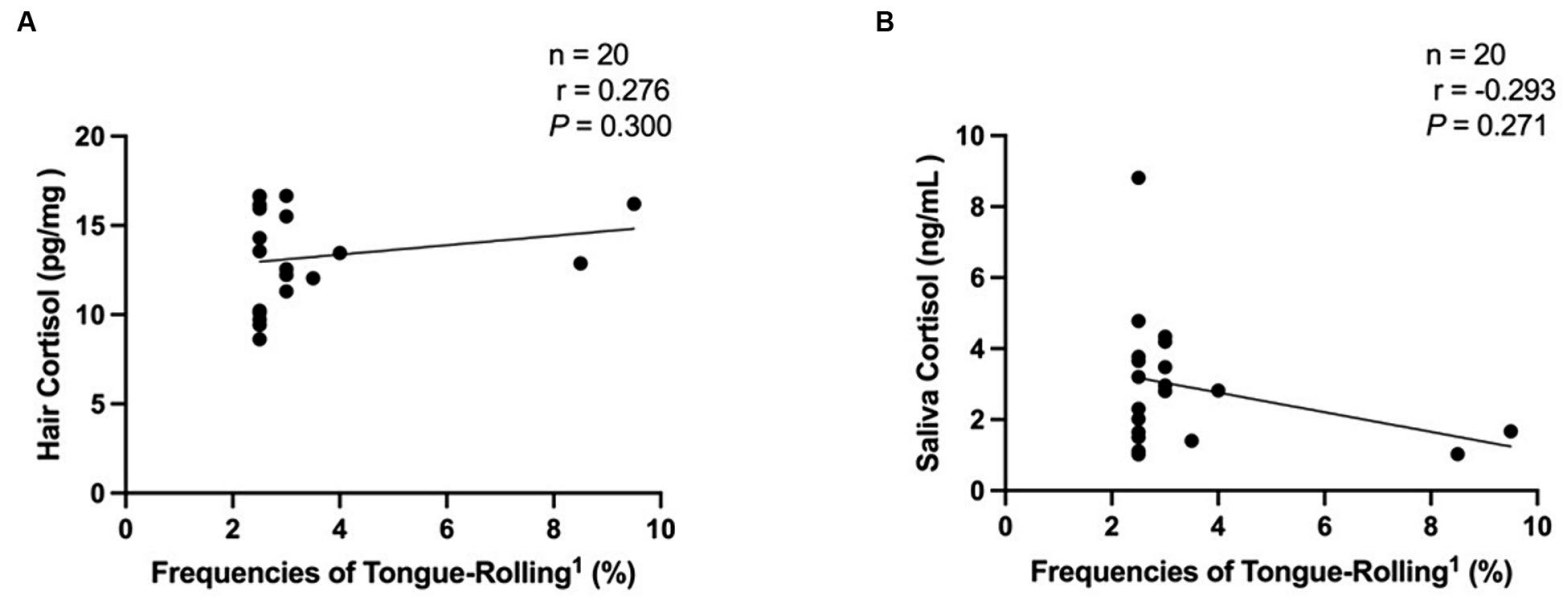
Analysis of the correlation between extreme tongue-rolling behaviour and hair and salivary cortisol concentrations in dairy cows. We selected cows with a number of tongue-rolling that was greater than 1.5 times the number in the first quartile as the cows with extreme tongue-rolling behaviour. Among all the tongue-rolling cows that were tested for cortisol levels in hair or saliva, 20 cows exhibited extreme tongue-rolling behaviour. **(A)** The analysis of the correlation between extreme tongue-rolling behaviour and hair cortisol concentrations. **(B)** The analysis of the correlation between extreme tongue-rolling behaviour and saliva cortisol concentrations. ^1^Frequencies of Tongue-Rolling: the percentage of the number of tongue-rolling expressions out of the total number of observations for cows exhibited extreme tongue-rolling behaviour to have cortisol concentrations, where the total number of observations is 200.

We then performed Spearman correlation analyses of the frequencies of tongue-rolling behaviour expression and hair and salivary cortisol concentrations. The number of cows with tongue-rolling behaviour was 180 out of 601 cows. As shown in Figure 4, there were no correlations between the frequencies of tongue-rolling expressions and hair cortisol (r = −0.046, *p* = 0.538) or between the frequencies of tongue-rolling expressions and salivary cortisol (r = −0.019, *p* = 0.800). Similarly, we retained the ETR group for Spearman correlation analyses with cortisol concentrations. Twenty cows remained and all had 5 or more occurrences of tongue-rolling. As shown in Figure 5, there were also no correlations between the frequencies of tongue-rolling expressions and hair cortisol (*n* = 20, r = 0.276, *p* = 0.300) or between the frequencies of tongue-rolling expressions and salivary cortisol (*n* = 20, r = −0.293, *p* = 0.271).

### 3.3 Lactation performance

**Figure 6:**
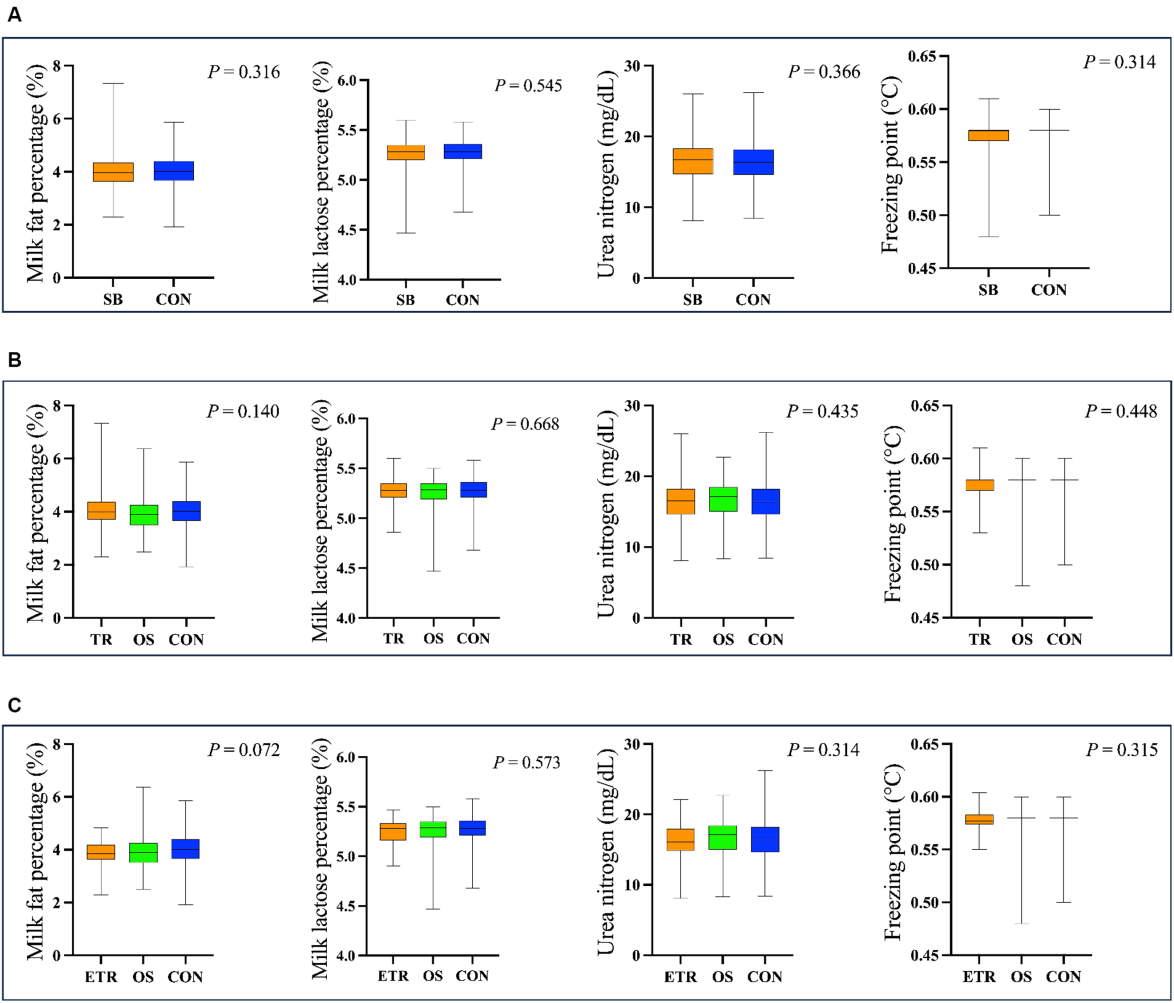
The results of milk fat percentage, milk lactose percentage, urea nitrogen and freezing point in different cows. **(A)** The cows were divided into SB group (The group of 337 cows with stereotypic behaviour) and CON group (The group of 425 cows without stereotypic behaviours). **(B)** The cows were divided into TR group (The group of 245 cows with tongue-rolling behaviour), OS group (The group of 92 cows with stereotypic behaviours except tongue-rolling) and CON group. **(C)** The cows were divided into ETR group (The group of 34 cows with extreme tongue-rolling behaviour. We selected cows with a number of tongue-rolling that was greater than 1.5 times the number in the first quartile as the cows with extreme tongue-rolling behaviour), OS group and CON group.

**Figure 7:**
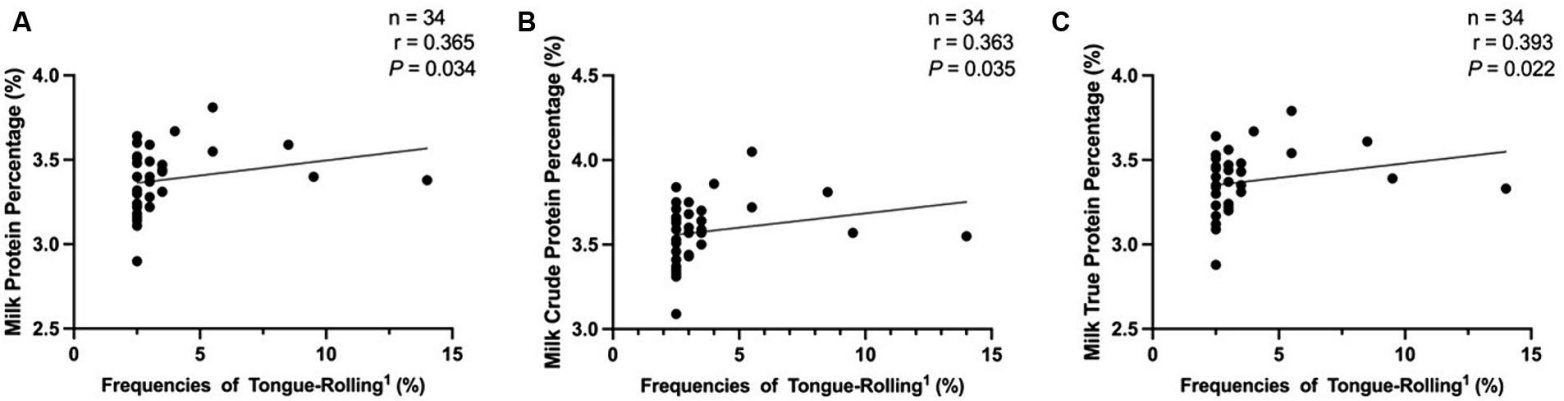
Analysis of the correlation between extreme tongue-rolling behaviour and milk protein rate, milk crude protein rate or milk true protein rate in dairy cows. We selected cows with a number of tongue-rolling that was greater than 1.5 times the number in the first quartile as the cows with extreme tongue-rolling behaviour. Among all the cows that collected lactation performance data, 34 cows exhibited extreme tongue-rolling behaviour. **(A)** The analysis of the correlation between extreme tongue-rolling behaviour and milk protein rate in dairy cows. **(B)** The analysis of the correlation between extreme tongue-rolling behaviour and milk crude protein rate in dairy cows. **(C)** The analysis of the correlation between extreme tongue-rolling behaviour and milk true protein rate in dairy cows. ^1^Frequencies of Tongue-Rolling: the percentage of the number of tongue-rolling expressions out of the total number of observations for cows exhibiting extreme tongue-rolling behaviour to have lactation performance data, where the total number of observations is 200.

In the analysis of lactation performance of cows, we obtained data on lactation performance of 762 cows. As shown in Tables 4–6, Figure 6, Supplementary Tables S1–S3, and Supplementary Figure S1, only when the extreme tongue-rolling behaviour was considered, the milk protein percentage, milk true protein percentage, and milk crude protein percentage of the ETR group cows were significantly lower than those of the NTR group (*p*<0.05, Table 6; Supplementary Table S3, including OS and CON group). Therefore, we performed Spearman correlation analysis on the frequencies of tongue-rolling expressions and lactation performance in cows with extreme tongue-rolling behaviour. As shown in Figure 7, there were correlations between the frequencies of tongue-rolling expressions and milk protein percentage (*n* = 34, r = 0.365, *p* = 0.034), milk crude protein percentage (*n* = 34, r = 0.363, *p* = 0.035), and milk true protein percentage (*n* = 34, r = 0.393, *p* = 0.022).

**Table 4:** The lactation performance in cows with stereotypic behaviours and normal cows (*n* = 762).

Items	SB^a^	CON^b^	SEM	*p*-value	*F*-value	df
*n*	337	425				
Milk protein percentage (%)	3.46	3.47	0.010	0.407	0.087	760
Milk crude protein percentage (%)	3.66	3.67	0.010	0.506	0.326	760
Milk true protein percentage (%)	3.45	3.47	0.010	0.467	0.092	760
Milk yield (kg/d)	32.17	32.82	0.261	0.215	0.125	760
Milk protein yield (kg/d)	1.11	1.14	0.010	0.136	0.180	760
Milk lactose yield (kg/d)	1.69	1.73	0.014	0.189	0.171	760
Milk fat yield (kg/d)	1.27	1.31	0.013	0.139	0.105	760
Milk crude protein yield (kg/d)	1.17	1.20	0.010	0.153	0.214	760
Milk true protein yield (kg/d)	1.11	1.13	0.010	0.146	0.214	760
4% FCM (kg/d)^c^	31.92	32.75	0.282	0.215	0.199	760

The “*n*” in the table heading represents the number of cows.

In this experiment, lactation performance data were collected from 762 dairy cows, so the sample size for Tables 4, 5 were 762.

a SB: The group of cows with stereotypic behaviours.

b CON: The group of cows without stereotypic behaviours.

c 4% FCMS (kg/d): 4% Fat corrected milk (kg/d) = 0.4 M + 15F, M: milk yield (kg/d), F: milk fat yield (kg/d).

**Table 5:** The lactation performance in cows with tongue-rolling behaviour, cows with other stereotypic beahviours (except tongue-rolling) and normal cows (*n* = 762).

Items	TR^a^	OS^b^	CON^c^	SEM	*p*-value	*F*-value	df
*n*	245	92	425				
Milk protein percentage (%)	3.47	3.44	3.47	0.010	0.463	0.772	761
Milk crude protein percentage (%)	3.67	3.64	3.67	0.010	0.507	0.680	761
Milk true protein percentage (%)	3.46	3.43	3.47	0.010	0.461	0.776	761
Milk yield (kg/d)	32.04	32.53	32.82	0.261	0.395	0.395	761
Milk protein yield (kg/d)	1.10	1.12	1.14	0.010	0.311	1.171	761
Milk lactose yield (kg/d)	1.68	1.71	1.73	0.014	0.352	1.045	761
Milk fat yield (kg/d)	1.27	1.27	1.31	0.013	0.329	1.114	761
Milk crude protein yield (kg/d)	1.17	1.18	1.20	0.010	0.341	1.078	761
Milk true protein yield (kg/d)	1.10	1.11	1.13	0.010	0.333	1.101	761
4% FCM (kg/d)^d^	31.89	32.01	32.75	0.282	0.342	1.076	761

The “*n*” in the table heading represents the number of cows. In this experiment, lactation performance data were collected from 762 dairy cows, so the sample size for Tables 4, 5 were 762.

a TR: The group of cows with tongue-rolling behaviour.

b OS: The group of cows with other stereotypic behaviours (except tongue-rolling).

c CON: The group of cows without stereotypic behaviours.

d 4% FCM (kg/d): 4% Fat corrected milk (kg/d) = 0.4 M + 15F, M: milk yield (kg/d), F: milk fat yield (kg/d).

**Table 6:** The lactation performance in cows with extreme tongue-rolling behaviour, other stereotypic behaviours (except tongue-rolling) and normal cows (*n* = 551).

Items	ETR^1^	OS^2^	CON^3^	SEM	*p*-value	*F*-value	df
*n*	34	92	425				
Milk protein percentage (%)	3.38^b^	3.44^ab^	3.47^a^	0.011	0.028	3.195	550
Milk crude protein percentage (%)	3.58^b^	3.64^ab^	3.67^a^	0.011	0.023	3.298	550
Milk true protein percentage (%)	3.37^b^	3.43^ab^	3.47^a^	0.012	0.021	3.255	550
Milk yield (kg/d)	32.39	32.53	32.82	0.303	0.904	0.111	550
Milk protein yield (kg/d)	1.09	1.12	1.14	0.011	0.545	0.803	550
Milk lactose yield (kg/d)	1.70	1.71	1.73	0.016	0.881	0.114	550
Milk fat yield (kg/d)	1.22	1.27	1.31	0.014	0.223	1.534	550
Milk crude protein yield (kg/d)	1.15	1.18	1.20	0.012	0.568	0.768	550
Milk true protein yield (kg/d)	1.09	1.11	1.13	0.011	0.525	0.840	550
4% FCM (kg/d)^4^	31.30	32.01	32.75	0.322	0.441	0.858	550

The “*n*” in the table heading represents the number of cows. In this experiment, lactation performance data were collected from 762 dairy cows. After only retaining the ETR, OS and CON groups of cows, 551 cows remained, so the sample size for Table 6 was 551.

^a,b^Means within lines with different superscript letters are significantly different (*p* < 0.05), means with the same superscript letter are not significantly different.

1 ETR: The group of cows with extreme tongue-rolling behaviour. We selected cows with a number of tongue-rolling that was greater than 1.5 times the number in the first quartile as the cows with extreme tongue-rolling behaviour.

2 OS: The group of cows with other stereotypic behaviours (except tongue-rolling).

3 CON: The group of cows without stereotypic behaviours.

4 4% FCM (kg/d): 4% Fat corrected milk (kg/d) = 0.4 M + 15F, M: milk yield (kg/d), F: milk fat yield (kg/d).

## 4 Discussion

In this study, tongue-rolling behaviour was confirmed to be the most prevalent stereotypic behaviour among dairy cows. The purpose of our research was to explore the relationship between tongue-rolling behaviour and stress as well as lactation performance. We initially analysed the proportion of tongue-rolling cows within the entire herd in relation to the lactation stage. Our findings indicated that as DIM increased, the number of tongue-rolling cows initially increased and then decreased. However, it appeared that tongue-rolling behaviour was not correlated with cortisol levels in the cows’ bodies. Only when cows exhibited a higher tongue-rolling frequency, the milk protein percentage, milk crude protein percentage and milk true protein percentage increased with the rising tongue-rolling frequency.

### 4.1 Prevalence of stereotypic behaviours in dairy cows

Stereotypic behaviours are frequent in dairy cows and occur at every stage of growth, including calves and lactating cows (10, 42). In a behavioural observational study of 8,158 cows, it was found that the incidence of tongue-rolling varied between parities (7). Therefore, first-lactation cows from a large dairy farm were selected for our experiment to exclude the effects of different parities. In our observations, tongue-rolling was found to be the stereotypic behaviour with the highest prevalence. This shows that tongue-rolling is the most common stereotypic behaviour in cows, which is in full agreement with the previous opinion (7, 10, 43–45).

After we calculated the proportion of cows with tongue-rolling behaviour at different stages of lactation, we found that the proportion of cows with tongue-rolling behaviour gradually increased from early to late lactation, and then gradually decreased after late lactation. Similarly, Robbins et al. (7) also found the relationship between the proportion of cows with tongue-rolling behaviour and the lactation stage. However, there may be multiple reasons for the decrease in the proportion of cows with tongue-rolling behaviour in late lactation (such as reduced feed intake, cows in late pregnancy). More experiments are needed in the future to explore the relationship between lactation and stereotypic behaviour.

Our study showed a higher occurrence of tongue-rolling compared to prior studies (45, 46). The variation in the results could be ascribed to the differences in the methods of observation. In contrast to other studies (7, 45, 46), our behavioural observations were of a longer duration, which allowed for the identification of a substantial number of cows with tongue-rolling behaviour.

### 4.2 Is stereotypic behaviour a way for cows to cope with stress?

According to our hypothesis, cows showing tongue-rolling behaviour should have lower hair or saliva cortisol concentrations than normal cows, but this was not the case. There were no differences in hair and salivary cortisol concentrations between TR and CON group. This may be because tongue-rolling behaviour was observed only once in more than 30% of all cows with tongue-rolling. However, there was still no difference in cortisol when only the ETR cows were retained, nor correlation between tongue-rolling frequency and cortisol for either the TR or ETR groups.

The study by Fureix et al. (15) analysed the relationship between blood or faecal cortisol and stereotypic behaviours in horses, and it was concluded that there was no significant relationship between stereotypic behaviour and cortisol concentrations. In addition, there have been similar findings of no relationship between stereotypic behaviour and cortisol concentrations in studies over the last decade (2, 21, 47, 48).

Hair cortisol is a validated measure of dairy cow’s stress state over a period of time (32–34, 49), however concentrations can be influenced by several factors. For example, high cortisol concentrations are seen in calves at 15 days of age and high hair cortisol concentrations in cows after calving (37). Birth and calving are difficult processes for animals and can cause severe stress reactions. In adult Holstein cows, the rate of hair growth is approximately 0.6 ~ 1 cm per month (50), with a complete molt every 3 months in animals (50). Therefore, 2 to 4 cm of hair sample reflects approximately 3 months of cortisol concentrations (51). In our experiment, the cows selected for sampling had lactation dates of 90 days or more to remove the effects of calving.

In summary, our results suggest stereotypic behaviour, particularly tongue-rolling may not be a way for cows to cope with stress. However, it is well known that stress is a complex process resulting from a non-specific response. The HPA axis is only the most typical of the many stress response pathways, in addition to the LC-NE axis, etc. (52). Therefore, other experiments may be needed at a later stage to detect other stress indicators, such as dopamine (DA), to explore the relationship between stereotypic behaviour and stress.

### 4.3 Stereotypic behaviour and lactation performance

It is well known that tongue movement promotes salivation. Tongue-rolling is also a form of tongue movement. Previous studies have shown that ruminant saliva is alkaline while the internal environment of the rumen is acidic (53). Prolonged tongue movement increases the production of saliva, which helps to neutralise stomach acid. The pH of the rumen environment influences the health of the organism and microbial growth (53–55).

In our experiment, cows in the ETR group had lower milk protein percentage than normal cows. Therefore, cows in the ETR group may have excessive rumen acidity that exceeds the appropriate rumen pH. Rumen microbial activity stagnates and bacterial protein synthesis is reduced. This results in lower milk protein percentage, crude milk protein percentage and true milk protein percentage in cows in the ETR group. In addition, the correlation between the frequencies of tongue-rolling expressions and milk protein rate, milk crude protein rate and milk true protein rate could indicate that as the frequencies of tongue-rolling expressions increased, the salivary secretion of the cows increased, the rumen acidity decreased and the microbial activity in the rumen gradually increased. Therefore, there was a positive correlation between the frequencies of tongue-rolling expressions and milk protein rate, milk crude protein rate and milk true protein rate.

From the DHI data of cows, we speculated that cows with tongue-rolling may suffer from rumen discomfort, which is relieved by highly expressing tongue-rolling behaviour. Bergeron et al. (56) suggested that oral stereotypic behaviours may be an indication of gastrointestinal discomfort. In the study by Sun et al. (45), the rumen pH of cows with tongue-rolling was lower than that of normal cows, and showed a tendency to decrease and then increase with increasing tongue-rolling behaviour. This also suggests that tongue-rolling behaviour may play a role in alleviating ruminal discomfort.

## 5 Conclusion

We combined hair or salivary cortisol and stereotypic behaviours to show the relationship between stereotypic behaviours and stress in terms of hair cortisol and salivary cortisol. According to our current data, tongue-rolling is unlikely to be a stress-coping strategy for cows, at least from a cortisol perspective, and this result may not support the “coping hypothesis.” However, the high frequency of tongue-rolling appears to have positive effects on lactation performance in dairy cows. Of course, more experiments are needed in the future to further explore the relationship between stereotypic behaviours and stress. However, from a production performance point of view, tongue-rolling is an indication of rumen discomfort in cows, and cows can alleviate their discomfort by expressing tongue-rolling behaviour frequently. In the dairy industry, we may be able to initially determine the rumen condition of cows by observing the number of tongue-rolling they express.

## Data availability statement

The original contributions presented in the study are included in the article/Supplementary material, further inquiries can be directed to the corresponding author.

## Ethics statement

The animal studies were approved by Animal Ethics Committee of the Chinese Academy of Agricultural Sciences. The studies were conducted in accordance with the local legislation and institutional requirements. Written informed consent was obtained from the owners for the participation of their animals in this study.

## Author contributions

CL: Data curation, Methodology, Writing – original draft. XC: Investigation, Visualization, Writing – review & editing. TF: Validation, Visualization, Writing – review & editing. XG: Conceptualization, Writing – review & editing.
