# Supplementary material for: Do cows with stereotypic tongue-rolling behaviour cope better with their environment?
Source: Front Vet Sci. 2024 May 22;11:1404539. doi: 10.3389/fvets.2024.1404539 (PMC11151878; doi:10.3389/fvets.2024.1404539)

Supplementary Material

# Supplementary Figures and Tables

## Supplementary Tables

**Supplementary Table 1.** The lactation performance in cows with or without tongue-rolling behaviour (n=762)

| **Items** | **TR^1^** | **NTR^2^** | **SEM** | **P-value** | **F-value** | **df** |
| --- | --- | --- | --- | --- | --- | --- |
| n | 245 | 517 |  |  |  |  |
| Milk protein percentage (%) | 3.47 | 3.47 | 0.010 | 0.813 | 0.124 | 760 |
| Milk crude protein percentage (%) | 3.67 | 3.67 | 0.010 | 0.703 | 0.231 | 760 |
| Milk true protein percentage (%) | 3.47 | 3.46 | 0.010 | 0.707 | 0.060 | 760 |
| Milk yield (kg/d) | 32.04 | 32.77 | 0.262 | 0.188 | 1.194 | 760 |
| Milk protein yield (kg/d) | 1.10 | 1.14 | 0.009 | 0.137 | 1.584 | 760 |
| Milk lactose yield (kg/d) | 1.68 | 1.73 | 0.014 | 0.111 | 1.818 | 760 |
| Milk fat yield (kg/d) | 1.27 | 1.30 | 0.013 | 0.225 | 0.116 | 760 |
| Milk crude protein yield (kg/d) | 1.17 | 1.20 | 0.010 | 0.149 | 1.699 | 760 |
| Milk true protein yield (kg/d) | 1.10 | 1.13 | 0.010 | 0.153 | 1.609 | 760 |
| 4% FCM (kg/d)^3^ | 31.89 | 32.62 | 0.283 | 0.226 | 0.831 | 760 |

The ‘n’ in the table heading represents the number of cows.

In this experiment, lactation performance data were collected from 762 dairy cows, so the sample size for Supplementary table 1 and Supplementary table 2 were 762.

^1^TR: The group of cows with tongue-rolling behaviour.

^2^NTR: The group of cows without tongue-rolling behaviour (include OS and CON).

^3^4% FCM (kg/d): 4% Fat corrected milk (kg/d) = 0.4M+15F, M: milk yield (kg/d), F: milk fat yield (kg/d).

**Supplementary Table 2.** The lactation performance in cows with only tongue-rolling behaviour, with both tongue-rolling and other stereotypic behaciours, with other stereotypic behaviours (except tongue-rolling) and normal cows (n=762)

| **Items** | **OTR^1^** | **TROS^2^** | **OS^3^** | **CON^4^** | **SEM** | **P-value** | **F-value** | **df** |
| --- | --- | --- | --- | --- | --- | --- | --- | --- |
| n | 194 | 51 | 92 | 425 |  |  |  |  |
| Milk protein percentage (%) | 3.46 | 3.48 | 3.44 | 3.47 | 0.010 | 0.647 | 0.552 | 761 |
| Milk crude protein percentage (%) | 3.66 | 3.68 | 3.64 | 3.67 | 0.010 | 0.673 | 0.513 | 761 |
| Milk true protein percentage (%) | 3.46 | 3.48 | 3.43 | 3.47 | 0.010 | 0.635 | 0.570 | 761 |
| Milk yield (kg/d) | 31.99 | 32.20 | 32.53 | 32.82 | 0.261 | 0.595 | 0.631 | 761 |
| Milk protein yield (kg/d) | 1.10 | 1.12 | 1.12 | 1.14 | 0.010 | 0.470 | 0.844 | 761 |
| Milk lactose yield (kg/d) | 1.68 | 1.69 | 1.71 | 1.73 | 0.014 | 0.692 | 0.713 | 761 |
| Milk fat yield (kg/d) | 1.27 | 1.28 | 1.27 | 1.31 | 0.013 | 0.526 | 0.745 | 761 |
| Milk crude protein yield (kg/d) | 1.16 | 1.18 | 1.18 | 1.20 | 0.010 | 0.500 | 0.789 | 761 |
| Milk true protein yield (kg/d) | 1.10 | 1.12 | 1.11 | 1.13 | 0.010 | 0.493 | 0.801 | 761 |
| 4% FCM (kg/d)^5^ | 31.84 | 32.07 | 32.01 | 32.75 | 0.282 | 0.536 | 0.728 | 761 |

The ‘n’ in the table heading represents the number of cows.

In this experiment, lactation performance data were collected from 762 dairy cows, so the sample size for Supplementary table 1 and Supplementary table 2 were 762.

^1^OTR: The group of cows only with tongue-rolling behaviour.

^2^TROS: The group of cows with tongue-rolling behaviour and other stereotypic behaviours.

^3^OS: The group of cows with other stereotypic behaviours (except tongue-rolling).

^4^CON: The group of cows without stereotypic behaviours.

^5^4% FCM (kg/d): 4% Fat corrected milk (kg/d) = 0.4M+15F, M: milk yield (kg/d), F: milk fat yield (kg/d).

**Supplementary Table 3.** The lactation performance in cows with extreme tongue-rolling behaviour and without tongue-rolling behaviour (n=551)

| **Items** | **ETR^1^** | **NTR^2^** | **SEM** | **P-Value** | **F-value** | **df** |
| --- | --- | --- | --- | --- | --- | --- |
| n | 34 | 517 |  |  |  |  |
| Milk protein percentage (%) | 3.38 | 3.47 | 0.11 | 0.018 | 5.270 | 549 |
| Milk crude protein percentage (%) | 3.58 | 3.67 | 0.011 | 0.014 | 6.420 | 549 |
| Milk true protein percentage (%) | 3.37 | 3.46 | 0.012 | 0.014 | 5.877 | 549 |
| Milk yield (kg/d) | 32.39 | 32.77 | 0.303 | 0.762 | 1.411 | 549 |
| Milk protein yield (kg/d) | 1.09 | 1.13 | 0.011 | 0.373 | 0.247 | 549 |
| Milk lactose yield (kg/d) | 1.70 | 1.73 | 0.016 | 0.716 | 0.123 | 549 |
| Milk fat yield (kg/d) | 1.22 | 1.30 | 0.014 | 0.184 | 0.242 | 549 |
| Milk crude protein yield (kg/d) | 1.15 | 1.20 | 0.012 | 0.377 | 0.225 | 549 |
| Milk true protein yield (kg/d) | 1.09 | 1.13 | 0.011 | 0.353 | 0.137 | 549 |
| 4% FCM (kg/d)^3^ | 31.30 | 32.62 | 0.322 | 0.325 | 0.044 | 549 |

The ‘n’ in the table heading represents the number of cows.

In this experiment, lactation performance data were collected from 762 dairy cows. After only retaining the ETR and NTR (including OS and CON groups) groups of cows, 551 cows remained, so the sample size for Supplementary table 3 was 551.

^1^ETR: The group of cows with extreme tongue-rolling behaviour. We selected cows with a number of tongue-rolling that was greater than 1.5 times the number in the first quartile as the cows with extreme tongue-rolling behaviour.

^2^NTR: The group of cows without tongue-rolling behaviour.

^3^4% FCM (kg/d): 4% Fat corrected milk (kg/d) = 0.4M+15F, M: milk yield (kg/d), F: milk fat yield (kg/d).

## Supplementary Figures

**Supplementary Figure 1.** The results of milk fat percentage, milk lactose percentage, urea nitrogen and freezing point in different cows. A: The cows were divided into TR group (The group of 245 cows with tongue-rolling behaviour) and NTR group (The group of 517 cows without tongue-rolling behaviour). B: The cows were divided into OTR group (The group of 194 cows only with tongue-rolling behaviour), TROS group (The group of 51 cows with tongue-rolling behaviour and other stereotypic behaviours), OS group (The group of 92 cows with stereotypic behaviours except tongue-rolling) and CON group. C: The cows were divided into ETR group (The group of 34 cows with extreme tongue-rolling behaviour. We selected cows with a number of tongue-rolling that was greater than 1.5 times the number in the first quartile as the cows with extreme tongue-rolling behaviour) and NTR group.


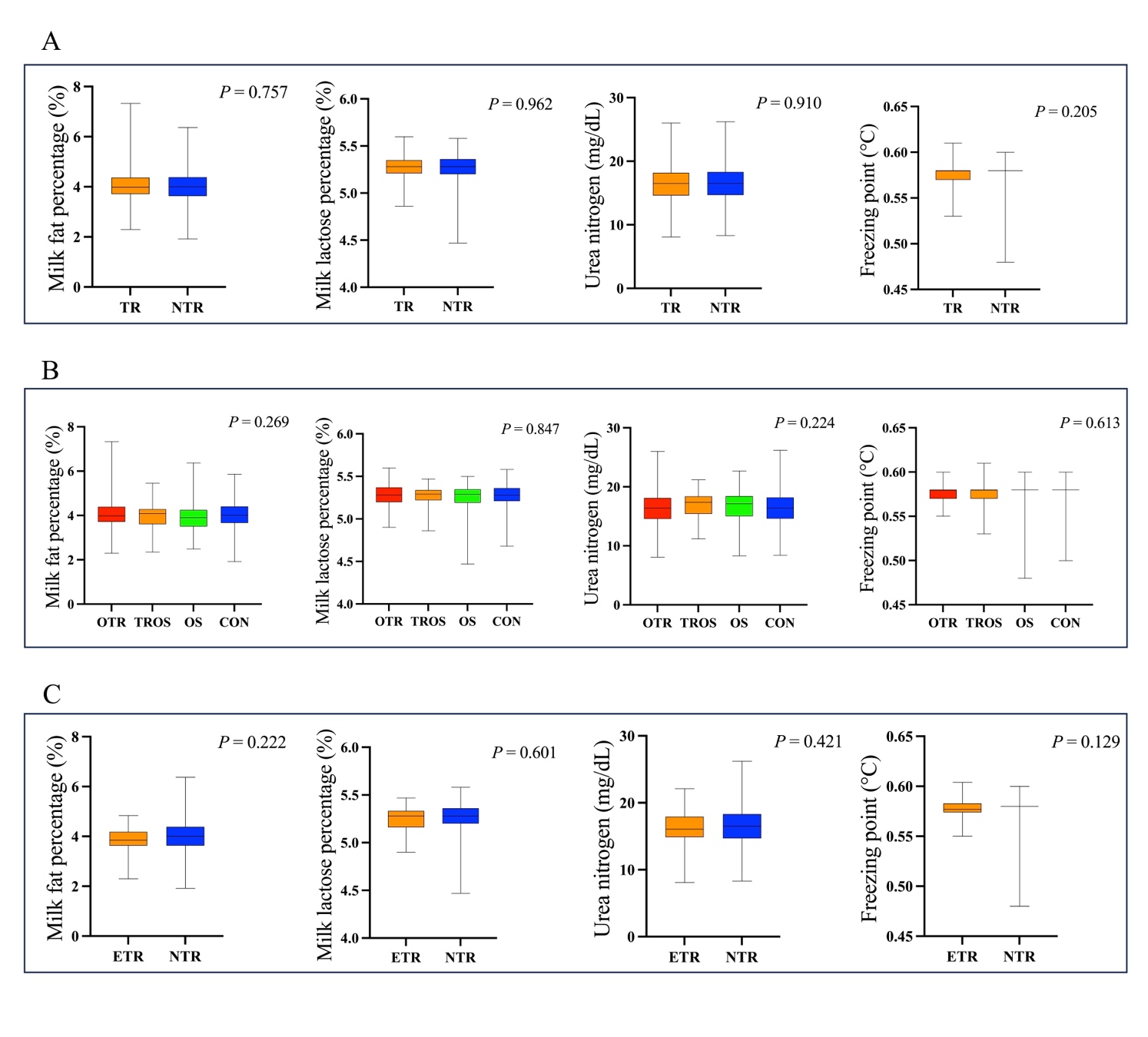

Supplement: Supplementary file 1 [file Data_Sheet_1.docx]
